# From fragmented care back to social medicine: European policy responses to the needs of complex patients

**DOI:** 10.3325/cmj.2023.64.143

**Published:** 2023-04

**Authors:** Aleksandar Džakula, Dorja Vočanec, Danko Relić

**Affiliations:** 1 Department of Social Medicine and Organization of Health Care, Andrija Štampar School of Public Health, University of Zagreb School of Medicine, Zagreb, Croatia; dvocanec@snz.hr; 2 Department of Medical Statistics, Epidemiology, and Medical Informatics, Andrija Štampar School of Public Health, University of Zagreb School of Medicine, Zagreb, Croatia

## The term “complex patients” in the European Union documents

Although the health care systems in the European Union (EU) member states are regulated at the national level, health and health care issues are constant EU priorities. The EU wants to preserve and continuously develop two key values: security and equity related to citizens' health (1). Equity in health and the related access to health care and universal coverage are by no means new topics. However, the systematicity and strength with which the EU approaches these problems have a positive trend. Bearing in mind these values and looking at the EU’s wider policies, the problem of complex patients is expected to be recognized as a priority. However, the term “complex patient” is rarely present in EU documents. One of the reasons is that the term came into global use only a few years ago (2). However, there are other reasons, perhaps much more significant.

## Fragmentation of care and hyperregulation

Due to the dominant medical model that views the patient through the lens of diagnosis, complex patients are separated into groups of patients with certain diseases. Such compartmentalization arises due to administrative and organizational reasons. However, it disperses the patients’ power of advocacy, and patients are locked in silos related to a disease or treatment option. In reality though, their needs are largely common. At first glance, a person with a hip fracture and a person with diabetic retinopathy have nothing in common. But, looking through the prism of functional ability in daily life and nursing care, they have a number of common needs. Only on top of these common needs, there comes a segment of care specific to the underlying medical condition (3,4).

There are pronounced differences between EU member states in citizens' access to health care, private health expenditure, quality and outcomes of care, etc. All the more, in some countries, there are considerable differences between individual regions or population groups (5,6). However, even the countries with the most accessible and effective systems are facing the fragmentation of care into particular, uncoordinated services (7-9). The most dramatic evidence of the cumulative effect of care fragmentation are poor care outcomes (eg, in oncology) combined with high treatment costs. This is also a counter-argument against the oversimplified thesis that the solution to better health care is more money. Research shows that a significant proportion of health spending only moderately improves care outcomes. In addition, part of the resources is not only spent on low-value health care, but literally goes to waste (10). It is a difficult task to integrate care within the health care systems, as well as to define and link it to the systematically monitored indicators (11,12). In addition to integration within health care itself, the EU emphasizes the interrelationship between health care and social care. This is best seen in the documents addressing population aging and long-term care (13). Perhaps, complex patients could be a long-sought-after subject for integrating care, and for introducing long-term structural changes in health care organization.

## European Pillar of Social Rights and vulnerable groups

One of the most precious values of the EU is the principle that defines health care as a universal human right, not a commodity. Because of many organizational or administrative barriers, there remains a gap between what is required as regards this right, and what is provided, but the general policies have a clear and unchanged direction. Health is seen as the fundamental social capital, and the basis of the economic and social development of the member states (14). This has turned out to be especially important during the recent crisis. In addition, the EU recognizes its role on the global scene and represents these values and integration of care through its global health strategy (15,16).

Complex patients could become a common denominator for all physically, mentally, or socially vulnerable people who are not able to meet their basic needs but do not belong to any specific group. This concept is a key framework through which people with complex conditions can request support. Without such categorization, many patients and caregivers remain an undefined set of individuals. In contrast, there are groups that form a subculture, such as migrants, LGBTQ+, or Roma, whose complex needs have been recognized. However, even within these groups, complexity remains for some, as their hidden axis of vulnerability. And it is precisely in relation to them that the principle of equity and fairness is practiced: to each as much as they need.

## Patients’ voices

Although care users and patients are recognized as partners throughout all the documents, their perspective and needs remain in the background. One of the attempts to change this, and to better recognize the complexity of needs, is the Roadmap toward inclusion of vulnerable groups' perspective within patients' organizations, a tool created by the European Patients' Forum (EPF). It states that “the main goal of this new initiative was to contribute towards EPF's strategic goals and core values and to promote the development of EU and national policies that tackle discrimination faced by patients in health and social care as well as in domains like education and employment” (17). New generations of EU documents recognize the move away from the strong authorities of systems and technologies toward the specific needs of individuals and caregivers, and the community in which they live. Therefore, their voice must be heard at the right time, in the right place, and in the right format. This is in line with the future vision of health care, which sees patients as protagonists of their own care (18).

## 2021-2027 Cohesion Policy and European Care Strategy

The most pronounced shift in EU policies is visible in the effort to move away from hospital-based to community-based and home-based care, especially when it comes to long-term care. This trend is supported through the financial mechanisms of the new cohesion policy, within one of its five priorities: a more social Europe (19). The reasons for this are manifold, but their meaning is particularly important in the segment of involving the patient and caregiver as partners, and the community as the primary setting that determines the complexity of needs. These models are challenged by increasing weakening of the family role and the creation of some new social networks. However, the meaning and role of the primary community can hardly be replaced by the industrial model of a medical institution. In September 2022, the European Commission presented the European Care Strategy to ensure quality, affordable, and accessible care services across the EU. The important novelty in the strategy is a comprehensive goal: to improve the situation for both care receivers and the people caring for them, professionally or informally (20).

## Entrustable Professional Activities

Since the lack of competence to work with vulnerable groups is often a barrier to the provision of quality and comprehensive care, the most common interventions are specifically designed education or empowerment programs for professionals. In recent years, changes have been noticed in this dimension of improving care for complex patients. Namely, the concept of entrustable professional activities (EPA) is increasingly mentioned. EPA should replace sets of competencies that often cannot be applied in real situations, with clearly defined abilities for health care professionals to comprehensively perceive and solve real situations (21). One of the stakeholders promoting this approach in education is the European Union of Medical Specialists, which brings together over 1.6 million medical specialists from 41 countries in the EU and its associated countries (22).

## Conclusion

After reviewing the EU political horizon, we can conclude that complex patients are well represented in EU documents, but hidden under different terms: vulnerable groups, people with rare diseases, people with mental health problems, excluded people, people with difficult access to health care services (prisoners, migrants…), etc.

An additional reason for their invisibility is the dominant top-down approach to shaping policies and strategic documents. With such an approach, it is not easy to predict or recognize in advance the combination of problems that will make up a complex case. Therefore, the policies use and describe the determinants, and care providers individually identify complexity in each specific case. Without the “complex patient” concept, these individual cases stay at the level of care provision or community and are not further recognized in official statistics to enter the documents in the new policy cycle.

Affirmation of the term and concept of “complex patient” can lead to a more significant integration and mobilization of resources and the improvement of care for patients and caregivers with the greatest needs. Therefore, EU policies, plans, and projects are an exceptional opportunity because they are aimed at the strategic allocation of resources based on solidarity, fairness, and other European values. Yes, EU policies and strategies are one of the key answers to the needs of complex patients.
